# The effectiveness of telegram‐based virtual education versus in‐person education on the quality of life in adolescents with moderate‐to‐severe asthma: A pilot randomized controlled trial

**DOI:** 10.1002/nop2.552

**Published:** 2020-06-28

**Authors:** Shiva Faraji, Sousan Valizadeh, Akbar Sharifi, Shahla Shahbazi, Morteza Ghojazadeh

**Affiliations:** ^1^ Department of Pediatric Nursing School of Nursing and Midwifery Tabriz University of Medical Sciences Tabriz Iran; ^2^ Iranian Center for Evidence‐Based Practice, Nursing and Midwifery School Tabriz University of Medical Sciences Tabriz Iran; ^3^ Tuberculosis and Lung Disease Research Center Tabriz University of Medical Sciences Tabriz Iran; ^4^ Department of Medical‐Surgical Nursing, Nursing and Midwifery School Tabriz University of Medical Sciences Tabriz Iran; ^5^ Iranian Center for Evidence‐Based Practice, Medicine School Tabriz University of Medical Sciences Tabriz Iran

**Keywords:** adolescent, asthma, distance education, e‐learning, mobile applications, quality of life

## Abstract

**Aim:**

In recent years, mobile applications have been developed for health education purposes. The aim of this study was to determine whether Telegram‐based virtual education versus in‐person education can be effective for improving the quality of life in adolescents with moderate‐to‐severe asthma.

**Design:**

A single‐blind randomized trial.

**Methods:**

Participants were 64 adolescents aged 12–19 years and were equally assigned randomly to group A (Telegram‐based education) and group B (in‐person education) during 22 June 2017–19 February 2018. The educational contents were similar for both groups. The Mini Pediatric Asthma Quality of Life Questionnaire was used prior to intervention and 5 weeks postintervention for both groups of adolescents.

**Results:**

A statistically significant increase was observed in the quality of life in both groups (*p *< .001). After controlling the quality‐of‐life scores, there was no statistically significant difference between the groups in terms of the mean score for the quality of life and its domains (*p *< .05).

## 1 INTRODUCTION

Asthma is a common chronic inflammatory disorder (Hockenberry, Wilson, & Wong, 2015), is one of the most prevalent health conditions around the world with an estimated 300 million currently diagnosed cases and predicted to reach 400 million by 2020 (Ohlmann, 2015), is a chronic respiratory disease among the children, is the primary reason for school absences, and is the third leading cause of hospitalization for children under the age of 15.

The prevalence of asthma in Iran was 8.9% [95% confidence interval (CI): 8.5–9.3] that was similar to other Asian and European countries (Fazlollahi et al., 2018). And among Iranian children, it varied from 1.26%–11.6% (Hassanzadeh, Basiri, & Mohammad‐Beigi, 2012). Despite the scientific and technological advances in medicine, asthma treatments have not reduced the yearly number of people dying from the disease.

An estimated 5 million children under the age of 18 in the United States are asthmatic (Cotton et al., 2012), and in addition to environmental factors, lifestyle and dietary regiment of fast foods among the adolescents have contributed to an increasing rate of asthma diagnosis (Braithwaite et al., 2014; Cepeda et al., 2017). Asthma has an effect on the physical, mental, social and developmental aspects of children's lives with a high financial burden on the families. Fear of an asthma attack limits daily activities, increases dependency on others and generates an undesirable quality of life (Zarei, Jahanpour, Alhani, Razazan, & Ostovar, 2014).

In recent years, healthcare providers and researchers have favoured using the quality of life as a reliable indicator for assessing the treatment success in asthmatic children (Payrovee, Kashaninia, Mahdaviani, & Rezasoltani, 2014). The quality of life is considered an important criterion for evaluating the progress of chronic diseases (Chen et al., 2011). The World Health Organization (WHO) has indicated that quality of life for asthmatic patients is a broad concept of physical health, mental status, personal independence, social relationships and environment (Payrovee et al., 2014).

For patients with chronic and incurable disorders, achieving and managing the best quality of life is the main goal (Nasmith, Kupka, Ballem, & Creede, 2013). Asthma is also a life‐threatening challenge with acute attacks and costly interventions. Asthma treatment and long‐term management require recognition of quality of life as an essential component of treatment plan (Chen et al., 2011).

### 1.1 Background

Patient education to improve their ability in administering self‐care is effective, also lowering the risks of developing complications and promoting the quality of life (Baraz, Zarea, & Shahbazian, 2017).

Some studies have shown that face‐to‐face education is more effective for patients’ respiratory self‐efficacy (Garrod, Marshall, & Jones, 2008). The social media (e.g. Facebook, Telegram) are increasingly visible in higher education settings as instructors look to technology to mediate and enhance their instruction and promote active learning for students (Crews & Butterfield, 2014; Tess, 2013).

Global healthcare systems could benefit from new models of care for patients with asthma. Telemedicine intervention has a great potential for educating patients with asthma. It facilitates remote access to health education and patient monitoring. Patients can receive life‐saving information via SMS, video conference, text messages, chats and emails (McLean et al., 2010). Health information technology can reduce the burden of chronic diseases such as asthma for patients and healthcare systems. Mobile health apps in particular could enable low‐cost, clinically efficacious interventions for asthma, asthma self‐management, improving a patient's quality of life through dissemination of educational materials and symptom monitoring tools (Tinschert, Jakob, Barata, Kramer, & Kowatsch, 2017).

Today, social networks such as Telegram, Viber, Instagram and WhatsApp are offering countless opportunities to inform and educate patients (Ebrahimpour et al., 2016).

Mobile‐based learning is a viable alternative for patient education in nursing (Kim & Park, 2019). In recent years, mobile applications have been developed for health education purposes, especially for asthmatic patients (Stukus et al., 2018). Telegram is a messaging mobile application similar to WhatsApp with the ability to create groups and supergroups as members or users to share large files such as documents, photographs and videos (Alabdulkareem, 2015). Educational intervention through Telegram app could be more appealing to adolescents with a higher success rate compared with other teaching methods of interventions.

According to the Lundy & Janes (2009), nurses are the largest group of healthcare providers. The nurses’ role in teaching through alternative methods was studied, and findings revealed the acceptance of chat apps by adolescents (Suter et al., 2015). But very few studies contributed data to quality of life (QoL) as outcome. Furthermore, no study has explored the use of Telegram‐based education (TBE) among the asthmatic adolescents to evaluate improvement in their quality of life. The purpose of this study was to determine whether Telegram‐based virtual education versus in‐person education can be effective for improving the quality of life in adolescents with moderate‐to‐severe asthma.

## 2 METHODS

### 2.1 Design of the study

This single‐blind randomized trial, pre‐ and post‐test equivalent control group design, was conducted to examine the effectiveness of a TBE versus in‐person education on the quality of life of adolescents with moderate‐to‐severe asthma. The registration code for this study on the clinical trial website is IRCT201704224613N23. We obtained institutional research ethics committee approval for this study to collect and analyse data from the Committee of Ethics at Tabriz University of Medical Sciences with number IR.TBZMED.REC. 1395.1309.

### 2.2 Participants

Adolescents diagnosed with moderate‐to‐severe asthma were recruited from a paediatric outpatient clinic specializing in respiratory diseases that serve as a referral centre affiliated with a public university in Tabriz, Iran, during 22 June 2017–2019 February 2018. The inclusion criteria consisted of being between 12–19 years, asthma diagnosis for over a month, willingness to be in the study and having a cell phone or computer, familiarity with the use of Telegram app on the mobile or computer, access to Internet and no prior participation in an educational programme.

Exclusion criteria included chronic diseases in addition to asthma and missing more than one training session.

Adolescents and their legal guardians signed an informed consent for voluntary participation with assurance for privacy and data confidentiality. The written informed consents were followed by a demographic survey questionnaire asking the individual's phone numbers to provide educational materials through Telegram app and help install the programme if absent for the training session.

Sixty‐four adolescents were equally assigned to group A (Telegram‐based education) and group B (control group) to receive in‐person education by a researcher who generated the random allocation sequence according to Rand‐List software and a 1:1 allocation ratio. Furthermore, for allocation concealment, the intervention content was written on paper and placed in sequentially numbered concealed envelopes.

### 2.3 Description of intervention

The educational contents such as subjects and structure were developed based on the outpatient requirements for asthmatic patients similar to previously published research (Zarei et al., 2014) and the National Asthma Education and Prevention Program guidelines (Rai et al., 2018; Williams, Schmidt, Redd, & Storms, 2003) for both groups. It consists of vignettes covering the basic pathophysiology of asthma, asthma symptoms, causes, environmental triggers, allergy agents, strategies to control and manage asthma, use of inhalers, how to use sprays, how to use a peak flow meter, sport, a travel guide to control asthma, nutrition and foods and medicines to asthma control (Castro & Kraft, 2008; Hockenberry et al., 2015). All the introduced topics were language‐ and age‐appropriate to adolescents.

After an expert review of the programme content, in‐person education and Telegram‐based sessions were offered. Content validity was established by paediatric professors from the Pediatrics and Internal Medicine Department, a professor from Tuberculosis and Lung Disease Research Center and a professor from the Pediatric Nursing Department with over 30 years of professional expertise.

A cell phone and phone number were taken from all participants. In group A, adolescents signed up for Telegram‐based education, the cell phone of participants or their companions regarding the existence of Telegram application was checked, and if needed, it was installed. They were taught how to use the Telegram application. High‐speed Internet was provided by the research team and used to avoid time lapses for downloading contents. They were received multimedia asthma educational contents by Telegram app to receive in form of video, photograph, voice recording and text messages. The intervention group was actively guided by experienced nurses. The 50‐min educational content on Telegram was divided into several parts, and after each episode, video, photograph, text and voice messages were sent to the participants to ensure that all participants had access to the educational contents to review and find answers to their questions. All follow‐up sessions in both groups began by reviewing and discussing questions on the previous contents.

In group B (in‐person education), educational sessions were held at the conference hall of the Nursing and Midwifery School for 32 participants in the control. Adolescents participated in five sessions once every three days for 50 min each, held by a researcher using PowerPoint^®^ and whiteboard within 2 weeks after entering the trial, followed by comments and discussions for another 15 min. At the beginning of each session, adolescents had an opportunity to ask questions and share additional content with the group on previous and new topics. The time interval was every 3 days to study the contents of the previous session and to satisfy the participants and as their requested.

The educational content was similar for both groups. Families and adolescents could call by telephone or Telegram chat with the case manager whenever they felt it necessary. For any communication delay or absence in either group, researchers used identifying date and education sessions to contact and follow‐up with the participants. As the delay or non‐attendance of the participants was expected at the appointed time, this issue was followed up and controlled by determining and emphasizing the date of the visit and contact by the researcher. In case of absence of more than one session, the person was excluded from the study.

### 2.4 Description of instrumentation

In a two‐part questionnaire, data were collected, where the first part completed during orientation session consisted of demographic information such as age, gender, asthma onset, family history of asthma, socio‐economic status and the place of residence. The second part included the Mini Pediatric Asthma Quality of Life Questionnaire (Mini PAQLQ) by Juniper et al. designed for paediatric patients between the ages of 11–18 years to determine the primary outcomes (Juniper, Guyatt, Cox, Ferrie, & King, 1999; Juniper et al., 1996). Data were collected in eight months starting from 22 June 2017–2019 February 2018, initiated during orientation session for both groups as baseline measurements (before) and 5 weeks after the intervention (post‐test).

The Mini PAQLQ Questionnaire has been used by many researchers in different countries such as South India, the United States and Spain (Galo et al., 2018; Hew et al., 2016; Macaden, John, & Christopher, 2017; Olsen, Stevens, Foster, & Hopp, 2015). Mini PAQLQ with 13 items focuses on three domains: activity limitation (three items), emotional function (four items) and asthma symptoms (six items). Response to each domain is scored on a standard scale of 1 means maximum impairment or worst state to 7 means no impairment, so high scores indicate better quality of life in Persian language (Payrovee et al., 2014). Reliability and validity of Mini PAQLQ were established in various studies among the adolescents with asthma in countries such as Greece and the United States (Grammatopoulou, Skordilis, Koutsouki, & Baltopoulos, 2008; Mahabaleshwarkar, Taylor, Tapp, & Dulin, 2016). In this study, the tool validity was tested in terms of content. The reliability was calculated using Cronbach's alpha coefficient value for all its domains were from 0.69–0.86 that provide an evidence for good internal consistency reliability.

### 2.5 Data analysis

The data were gathered at two time periods and were analysed by SPSS version 21, and the comparability of groups was examined using the chi‐square test and independent‐sample *t* test. The effect of intervention was described by the change in quality‐of‐life score of adolescents by comparing the baseline obtained before the intervention and after 5 weeks of virtual education through the analysis of covariance (ANCOVA), Mann–Whitney and paired‐sample *t* test, as appropriate. *p*‐value of .05 was considered as statistically significant. Data Analyzer was blinded to the data files and study process. Patients were not blind to the randomization process. Sample attrition occurred in groups due to withdrawals from the education programme at 6% rate, and we analysed a sample of 60 participants.

## 3 RESULTS

We found no statistical significance between the two groups at baseline demographic characteristics and the onset of asthma (*p *> .05) (Table 1). Also as presented in Table 2, there was no statistical significance in the total quality‐of‐life scores in both groups and their pre‐test (*p *= .90). However, after five weeks of intervention the quality‐of‐life scores increased in both groups (*p *< .001). Despite an increase in the post‐test quality‐of‐life score according to the ANCOVA results, there was no statistical difference in the mean scores for the quality of life after the intervention (*p *= .38).

**Table 1:** Distribution of demographic information of adolescents in two groups

Variable under consideration		Group A (*n* = 30)	Group B (*n* = 30)	*p* *
Severity of asthma *n* (%)	Moderate	25 (83.3)	26 (86.7)	1
	Severe	5 (16.7)	4 (13.3)	
Distribution of birth rate *n* (%)	1	16 (53.3)	16 (53.3)	0.51*
	2	10 (33.3)	7 (23.3)	
	3	4 (13.3)	7 (23.3)	
Economic status *n* (%)	High	0 (0.0)	4 (13.3)	0.13*
	Moderate	17 (56.7)	16 (53.3)	
	Low	13 (43.3)	10 (33.3)	
Living area *n* (%)	Tabriz city	23 (76.7)	22 (73.3)	0.77*
	Suburbs	7 (23.3)	8 (26.7)	
Gender *n* (%)	Female	8 (26.7)	14 (46.7)	0.11*
	Male	22 (73.3)	16 (53.3)	
History of asthma in family *n* (%)	Yes	5 (16.7)	5 (16.7)	1*
	No	25 (83.3)	25 (83.3)	
History of asthma in the family	Yes	10 (33.3)	7 (23.3)	0.39*
	No	20 (66.7)	23 (76.7)	
Age in years, *M* (*SD*)		16.97 (1.79)	16.67 (2.43)	0.59**
Duration of asthma (month) Median, 95% CI*		36 (3–132)	48 (3–120)	0.48**

Note

Group A: Telegram‐based education; Group B: in‐person education.

* Chi‐square test.

** independent‐sample Student's *t* test.

*** Mann–Whitney test.

**Table 2:** Mean and standard deviation of quality‐of‐life scores before and after the intervention in the groups

Quality of life	Group A (*n* = 30)	Group B (*n* = 30)	Comparison of the groups *p* **	PANCOVA**	Effect Size(η)
Before	4.26 (0.95)	4.33 (1.16)	.80		0.014
After	4.81 (0.75)	4.99 (0.97)	.43		
Differences (%95 CI)	−0.55 (−0.79, −0.31)	−0.66 (−0.94, −0.37)		0.37	
*p* *	<.001	<.001			

* Paired‐sample *t* test.

** independent‐sample *t* test.

*** Covariance test.

According to Table 3, all of the three sub‐scale scores for the quality of life, which consisted of “Activity,” “Symptoms” and “Emotion” were similar in both groups before the intervention (*p *> .05) and improved after the intervention (*p* < .001). However, the ANCOVA test results after the score control for pre‐test showed no statistically significant difference between the means for three specific sub‐scale domains at post‐test in both groups and after the five weeks of intervention (*p *> .05).

**Table 3:** Mean and standard deviation and variations of quality‐of‐life scores over time in all the domains in the groups

Dimensions of quality of life	Time	Group A (*n* = 30)	Group B (*n* = 30)	Comparison of the groups *p* **	PANCOVA**	Effect Size(η)
Activity limitation	Before	4.46 (1.30)	4.41 (1.33)	.89		0.026
	After	5.02 (0.90)	5.09 (0.07)	.80		
	Differences (%95 CI)	−0.56 (−0.81, −0.31)	−0.67 (−0.40, −0.95)		0.22	
	*p* *	<.001	<.001			
Symptoms of asthma	Before	3.78 (1.05)	3.72 (1.22)	.82		0.007
	After	4.48 (0.91)	4.57 (0.98)	.74		
	Differences (%95 CI)	−0.70 (−1.02, −0.38)	−0.85 (−1.19, −0.51)		0.52	
	*p* *	<.001	<.001			
Emotional function	Before	4.53 (1.26)	4.85 (1.64)	.60		0.002
	After	4.93 (1.11)	5.31 (1.45)	.54		
	Differences (%95 CI)	−0.39 (−0.06, −0.47)	−0.45 (−0.84, −0.06)		0.74	
	*p* *	<.005	<.018			

* Paired‐sample *t* test.

** independent‐sample *t* test.

*** Covariance test.

## 4 DISCUSSION

This is the first study where Telegram‐based education has been used to improve the quality of life in adolescents with asthma. Health education is an important part of paediatric asthma management (Rai et al., 2018; Williams et al., 2003), and TBE can equally be an effective approach compared with in‐person education for improving the quality of life for adolescents diagnosed with moderate‐to‐severe asthma.

Researchers considered the mean difference = 0.5 in Juniper's Asthma Quality of Life Questionnaire and determined that patient education had a clinically important role for improving the quality of life (McLean et al., 2010). Other studies have shown consistent results similar to our findings (Ahmed et al., 2016; Chan, Callahan, Sheets, Moreno, & Malone, 2003; O'hara, Vethanayagam, Majaesic, & Mayers, 2006; Tavakoli, Alipouran, & Zarei, 2018). However, no other study has examined the effects of offering patient education through Telegram for asthmatic adolescents. For diabetic patient education, Tavakoli et al., (2018) showed that educational messaging through Telegram could improve the perception constructs by using the health belief model (HBM) and emphasizing the self‐care behaviours. Similarly, O'hara et al., (2006) showed that Internet‐based education for asthma management can reduce the need for emergency intervention and stabilizing control, even when travelling to another location. McLean et al., (2010) reviewed 21 research articles in a wide range of technologies aimed at the impact of distance learning and reported that virtual interventions did not clinically improve the QoL for asthmatic patients, and these results show clinical improvement in scores of two domains for the QoL (activity limitation and asthma symptoms) and do not include the scores of emotion domains in two groups. These results are consistent with findings by Nair, Nair, & Sundaram, (2014) showing no statistically significant change in the emotional domain of Mini PAQLQ in children. In another systematic review of clinical trials published in 2015, Flodgren et al. examined effect of telemedicine (TM) on professional functions and healthcare outcomes. From 93 eligible randomized controlled trials reviewed, only five studies were focused on asthmatic patients. They found that TM use had no different effect on the QoL for 825 patients with asthma. For patients with heart failure, their health management outcomes showed the same results for TM, face‐to‐face care or through phone care (Flodgren, Rachas, Farmer, Inzitari, & Shepperd, 2015).

Ja Yun Choi et al. concluded innovative education methods may be needed to improve and to maintain pulmonary function, symptom control and asthma knowledge and health‐related quality of life of poorly compliant adult Korean patients with asthma (Choi & Cho Chung, 2011).

Regarding self‐directed Internet‐based education, researchers found the same effectiveness in a small group of students learning about the inhaler use techniques compared with the traditional patient education (Toumas, Basheti, & Bosnic‐Anticevich, 2009). The multimedia education increases knowledge and self‐efficacy for parents of children with asthma (Zarei et al., 2014), and researchers found a statistically significant difference on the effects of education for using peak flow meter and a follow‐up via SMS to control asthma (*p *= .002) (Pedram Razi, Piroozmand, Zolfaghari, Kazemnejad, & Firoozbakhsh, 2013).

In this study, we found the PAQLQ mean scores in all domains and for both groups were significantly improved. But, despite overall improvement in PAQLQ, there was equally improved PAQLQ for asthmatic adolescents in three domains in both groups. Although no study has directly compared the effect of TBE on the PAQLQ in adolescents with moderate‐to‐severe asthma, our study has addressed whether TBE can provide additional benefit over in‐person education as one of the most common educational interventions. The findings of this study are generalizable in locations, where people have access to the Telegram app and know how to use instant messenger.

### 4.1 Limitations

The study was completed in the fulfilment of a master's degree thesis and hence limited the sample size. Small sample size also affects the reliability of the study's results. So, to further generalize the findings, sampling with a large sample size was recommended.

## 5 CONCLUSION

Improving the quality‐of‐life scores in all domains for both groups revealed that both methods of education equally improved the QoL scores for adolescents with moderate‐to‐severe asthma. Therefore, face‐to‐face education as a traditional method still has a special value in health education offered by nurses and virtual education is a modern teaching method with features such as interactability and opportunity for being used in any place at any time most suitable for the patients and healthcare providers especially in paediatric wards. Nurses have a statistically significant role in providing patient education, and paediatric nurses have a unique opportunity for providing in‐person and Telegram‐based education to improve the quality of life for adolescents with asthma. The advantages of using these new methods to educate adolescents with asthma could be further investigated.

## RESEARCH ETHICS COMMITTEE APPROVAL

The approval code of ethics for this study is IR.TBZMED.REC.1395.1309.

## CONFLICT OF INTERESTS

The authors declare that there is no conflict of interest.

## AUTHOR CONTRIBUTIONS

SV, ShF and ShSh: Study design; ShF: Data collection; ShF and MG: Data analysis; and ShF, SV, ShSh and ASh: Drafting of the manuscript.
